# Puerperal Convulsions

**Published:** 1890-04

**Authors:** S. H. Stout

**Affiliations:** Cisco, Texas, Ex-Surgeon and Medical Director of the Hospitals of the late Confederate Army of Tennessee


DANIEL'S

### Texas Medical Journal.

A Representative Organ of the Medical Profession, and an Exponent of Rational Medicine; devoted to the Organization, Advancement and Elevation of the Profession in Texas.

PUBLISHED MONTHLY.—SUBSCRIPTION $2.00 A YEAR.

Vol. V. AUSTIN, APRIL, 1890. No. 10.

### Original Articles.

CONTRIBUTED EXCLUSIVELY TO THIS JOURNAL.

*The Articles in this Department are accepted and published with the understanding that we are not responsible for, nor do we indorse the views and opinions of the writers by so doing.*

For Daniel's Texas Medical Journal.

### PUERPERAL CONVULSIONS.

BY S. H. STOUT, A. M., M. D., LL. D., CISCO, TEXAS,

Ex-Surgeon and Medical Director of the Hospitals of the late Confederate Army of Tennessee.

TWICE before the date of this writing I have published a paper on the subject of Puerperal Eclampsia.

The first was communicated to the Nashville Medical Journal, several years before the late civil war. To this I have not present access. In it I gave publicity to the theoretical views of the pathology of this terrible complication of the puerperal state, which I reiterated in a "Report to the Section on Gynecology for the Seventh Congressional District," to the Medical Association of Georgia, and published in the volume of its Transactions for the year 1881.

To the date of writing that report I had encountered in my practice six cases of puerperal convulsions, all of which recovered as rapidly and as fully as the average puerperal patient, who has had no complication during parturition. Of these cases five occurred in my practice in Giles county, Tennessee, and one in Roswell, Georgia. My then recorded cases of childbirth, at full term, summed up nine hundred and eighty-eight. At that time eclampsia had complicated about six-tenths of one per cent.

Since that date I have encountered, in my own practice, two additional cases. The sum of all my cases of childbirth, at full term, being to the present time eleven hundred and eleven; the per centum of puerperal convulsions in my practice is now seven-tenths of one per cent.

In addition to the above, in consultation with my friend, Dr. C. S. Vance, of Cisco, I aided in the treatment of a case occurring in his practice. Thus making in all nine cases I have treated and aided in treating during an experience of nearly forty-two years as an active practitioner.*

In every one of these nine cases, copious blood-letting as the foundation of the treatment, was resorted to. My happy experience has vindicated my practice, and what I consider sound views of the pathology of puerperal eclampsia, have emboldened me to resort to venesection without stint as to quantity of blood drawn, until my patients have given evidence of the impressibility of the nervous system by anodynes, anæsthetics and nauseants.

Of the nine cases of this complication of the puerperal state I have treated and aided in treating, case one was a primipara, whose first paroxysm of convulsions occurred before any perceptible

*You were in error, Mr. Editor, when you stated that I have been a practitioner for half a century. It was exactly fifty years on the 5th of October, 1889, since I began the study of medicine.

symptoms of labor. Her "time was up," and I suspected that the convulsions were excited by the pains of labor. I bled the patient profusely, letting by estimation, (I did not have time to accurately measure it,) not less than thirty-two ounces of blood, before she could be impressed by anodynes, nauseants or anti-spasmodics. After becoming impressible by these, the labor proceeded naturally, a living healthy infant being born, without instrumental aid, after the lapse of about sixteen hours after I reached her bedside.

The second case was a multipara, and the third a primipara. I was called to both these cases from twelve to twenty hours after their delivery by ignorant widwives. They had walked out of their cabins to evacuate their bowels. In both cases I let blood profusely, until the desired impressibility by medicines was secured. Neither of them had had an abdominal bandage applied. In both I found the womb toppled over into the iliac region.

In cases four, six, seven and eight the convulsions first occurred after the child's head had impinged upon the perineum; in case five the first paroxysm occurred just after the body of the child was delivered. In case nine (my friend Dr. Vance's), the first convulsion occurred four or five hours after successful delivery of twins--a normal case of duplicate parturition.

Case two I delivered with the aid of a large spoon, used as a vectis. Cases six, seven and eight I delivered with the forceps.

The study of these cases indicates that Nos. 1, 3, 4, 6, 7 and 8 were primiparæ; that 2, 5 and 9 were multiparæ. No. 1 exhibited the first symptoms just as labor commenced. In this case the labor pains were the exciting cause. In Nos. 4, 6, 7 and 8, the exciting cause was the pain produced by the impinging of the fœtal head upon the perinæ of primiparæ. Cases Nos. 2 and 3 were excited by misplacement of the fundus of the uterus into the iliac region. In case 4 the exciting cause was the rapid passage of the shoulders of the child through the soft parts; and in case nine, probably laughter and two much disquiet around the patient's bedside.

In none of the cases was the blood or urine tested chemically.

Hence I do not know how much urea may have been in the blood, or how much or how little of it was discharged from the bladder.

I have never considered it important to rational treatment of such cases to ascertain whether uræmic poisoning existed or not. If I had so thought, I have never had time to make the necessary test.

That the mechanical pressure of the gravid uterus often abnormally affects the functions of the kidneys and their synurgical organs, and indeed of all the viscera, blood vessels, and nerves of the abdominal and pelvic regions, is obvious to every thoughtful and intelligent practitioner.

The exceptional impressibility of the nervous system of puerperal patients to morbific causes, is patent to every one who has frequented the lying-in chamber. Throughout the whole period of gestation abnormities of the nervous system, of the blood in circulation, and of viscera, especially those of the abdominal and pelvic regions, force themselves upon the attention of the medical practitioner. Abnormities of the nervous and vascular systems to which reflex action often gives alarming expression, are frequent sources of anxiety, not only because of the gravity of the symptoms, but also because they often obscure the diagnosis and wrap in mystery the ætiology of disease and its pathological consequences.

During the period extending from puberty to the menopause, the female organism in the state of health, is preparing to nourish a fœtus, or is actually nourishing one in the womb, or while lactating, is nourishing an infant.

If an ovule fails to lodge in the uterus or without it, because of its non-fecudation, the surplus of blood in the system not demanded for the nourishment of a second being, is thrown off in the form of menstrual fluid. If it lodges and takes root, the instinct of the organism, *pari passu*, with the demands of the growing fœtus, directs the supply of the requisite quantity of nutritious blood. If at any time the supply is greater, that is, needful, plethora, with its attendant symptoms and frequent baleful consequences, often becomes pronounced. And vice versa, if anemia, or a low state of vitality from any other cause, exists, in the unimpregnated, we meet with amenorrhœa, and iu the impregnated with abortion, in which we often find an emaciated fœtus; and generally in the syphilitic mother, a diseased placenta.

As the gravid uterus enlarges, especially in primiparæ, the mechanical pressure upon the abdominal and pelvic vicera (even sometimes obstructing the free movements of the heart and lungs), often provokes the abnormities of function of the kidneys, the intestines, the bladder, the respiratory and cardiac apparatus, the vascular circulation, and as a consequence of these, abnormal nervous symptoms that greatly inconvenience the patient, and sometimes indicate morbific factors, which, if not defeated of their baleful influence, may lead to fatal results.

The obstructions to the normal performance of the functions of the human organism, or any part of it, produced by this mechanical pressure, are of daily observation, not only when caused by a gravid uterus, but also by tumors of abnormal hystology, both benign and malignant.

About forty-six years ago, the substance known as *kiestine* found floating on the urine of certain females, was regarded by some reputable authorities as a certain indication of pregnancy, and many medical men were ready to go into court and swear that its presence in the urine of a female was a certain sign of pregnancy. But it has often been found floating on the urine of the hysterical and the chlorotic. According to Lehman, "the layer of kiestine is merely a formation of crystals of triple phosphate and fungoid and confervoid growths, which takes place when the urine becomes alkaline." [See Raeslee's Hystology, p. 88.]

In the pregnant woman the kidneys often abnormally perform their functions, because of a state of asthenia produced by mechanical crowding of those organs. So, too, in chlorosis and hysteria, there is often an asthenic condition of the kidneys. Hence, too, the appearance of kiestine floating on the urine of the chlorotic and the hysterical.

Who that has ever practiced obstetrics intelligently, has not met with various abnormities in the urine of pregnant and lying-in women, and rationally attributed them mainly to mechanical pressure upon the kidneys, bladder etc.? That it should often occur, that the normal amount of urea is not excreted by the kidneys of pregnant women, is no marvel to any practitioner of experience, because of the blood stasis in the vessels of those organs produced by mechanical pressure. Every intelligent practitioner is in the habit of recommending the recumbent posture, with the thighs flexed upon the body, to relieve that pressure, and as a means of overcoming, wholly or in part, the abnormities due to it.

The existence of urea in the blood of a pregnant female, and its non-elimination, may reasonably be looked for in many cases; indeed, in more cases than are complicated by eclampsia, either before, at the time of, or after parturition. Its presence in the few cases of puerperal eclampsia in which it has been detected, by no means proves that it is the sole predisposing or prime cause of this appalling complication of child bearing. If, *per se*, it is the predisposing cause of the eclampsia, why does not eclampsia occur oftener in pregnancy and parturient women, since in almost every case of pregnancy there is, at some time during gestation, parturition, and within the month after the latter, some perceptible evidences of abnormal functional action of the kidneys, and per consequence, of the bladder, as when the sandy products of the former irritate the latter, especially its neck? The presence of urea in the blood, when it appears therein, is to be accounted for as the consequence of diseased nutrition or deranged renal function.

Why is it that we often find diseased nutrition and deranged function in pregnant and parturient women? I have already suggested mechanical pressure as an important factor in the production of diseased nutrition and deranged function.

But preceding this, and often accompanying it, is another factor, in my opinion far more potent in the production of renal and cystic, gastric, enteric, pulmonic, cardiac, spinal, and cerebral abnormities of function. This is a failure of the organic instinct to weigh the amount of plastic and nutritious blood necessary to the normal support of mother and fœtus, and retain in the vascular system of the mother only the quantity needed therefor.

When the organic instinct thus fails to mete out the proper quantity of nutrition, or plastic blood, plethora follows. Plethora is incompatible with normal nervous action. Hence, in the obstetric patient nervous disturbances caused by it, more or less grave, are phenomena of frequent observation.

If the organic instinct retains normal control over the quantity and quality of the blood in the event of the non-fecundation of an ovule, the surplus of blood in the female is disposed of by a normal menstrual flux.

Dysmenorrhœa and suppressio mensium in robust subjects, are characterized by numerous and varied nervous abnormities, which are often of great gravity, and are most dangerous when the suppression is sudden and complete. Many a grave is occupied by blooming, robust maidens, hurried thither by a single act of imprudent exposure to sudden changes of temperature, in obedience to the demands of poverty, society, and the churches, at times of religious excitement.

Is it not irrational, then, to ignore the liability to failure of the organic instinct to proportion the quantity of plastic blood to the joint needs of the mother and her fœtus at any stage of gestation, during parturition and until the full establishment of lactation and the perfect involution of the uterus? The lochial discharge is a normal one, in part disposing of the surplus of blood remaining in the mother's reproductive and associated organs, no longer needed to nourish the fœtus in utero. The nervous and vascular abnormities consequent upon a sudden or a too early suppression of a lochia, are known to all familiars of the lying-in chamber.

Now the reader may inquire, "To what does this resume of facts and theory lead?" The answer is, to the vindication on rational theoretical grounds of what has in my experience always been successful practice in the treatment of puerperal eclampsia, which, unforunately, under the influence of flippant and unphilosophical lecturers and writers has fallen into culpable desuetude.

Plethora is often so grave as to partially paralyze every function,—sometimes so profound as to completely paralyze the vaso-motor nerves, whose normal influence is necessary to the continuance of the nutrition and even of the life of the patient. Sometimes the veins are so distended by plethora as to provoke therein blood stasis so complete that embolism, effusion of serum, through their walls, or rupture of their coats, permitting the escape of blood into the surrounding tissues, may occur. Plethora prevents absorption of nutriment and medicines. It more or less impairs the impressibility of the patient's nervous system by any kind of ingesta. It produces a feeling of feebleness on the part of the patient, which is too often mistaken by the practitioner and patient for a condition that forbids depletion, and seems to indicate the use of stimulants,—often a fatal error in practice.

In cases wherein puerperal eclampsia is threatened, as indicated by dizziness so profound as to obscure the intellect, it has always been my practice to let blood until the head symptoms are decidedly palliated before resorting to anæsthetics or anti-spasmodics. So too, when eclampsia suddenly and unexpectedly appalls the by-standers, the first thing I do is to bleed, (profusely, if necessary,) until I think the patient's impressibility by appropriate medication is assured. If the trial of remedies convinces me that impressibility has not been secured, I resort to bleeding again and again. The quantity of blood which a patient, laboring under puerperal eclampsia, can part with, without endangering life, or interrupting rapid recovery, is sometimes as great as would cause a horse to stagger, if let from his veins.

The quality of blood let in all of my cases was remarkable for the proportion of its crassamentum, which, so far as I was able to perceive, remained unchanged to the last. In every case the recovery of the patient was as rapid and complete as in ordinary cases of parturition, and in none was there any evidences of anæmia.

The extraordinary quanity of crassamentum in the blood drawn from patients affected with puerperal eclampsia, is accounted for by the theoretical views above expressed, which were published to the world in the paper I have made mention of in the initial paragraph of this communication. For these views I claim originality. If any one had previously publicly given utterance to them, I am ignorant thereof.

Admitting that those views are false wholly or in part as touching the failure of the organic instinct to regulate the quantity and quality of nutritious blood constituents needed by both mother and child, there are potent considerations for resorting to blood-letting as the means of assuring successful therapeusis in the treatment of eclampsia with anæsthetics, anti-spasmodics, nauseants and anti-plastics internally, and anæsthetics by inhalation.

Blood-letting in plethora, that has slowed the heart's contractions and diminished the frequency of the pulse, encourages more rapid movement of the blood in the veins, and consequently relieves the laboring heart, and promotes the absorption of ingesta, whether nutriments or medicines.

Admitting for the sake of argument, that the now fashionable theory that uræmia is the sole cause of puerperal eclampsia, is true, how absurd is the practice based upon it that prompts the practitioner to stand by the side of the eclamptic woman persistently refusing to let out by venesection any part of it, and thus prepare the way for the absorption of rationally indicated medicines?

A writer in the University Medical Magazine (Nov., 1880), reviewing Vol. II. of "American System of Obstetrics," uses the following language:

"In his account of the etiology of 'Eclampsia,' Professor Parvin seems to 'count it a bondage to fix a belief.' At the present day there is no occasion for 'affecting free-will in thinking' upon this topic, to the injury of the patient. Puerperal convulsions are in the very large majority of cases the expression of uræmia, conditioned upon functional or organic disease of the kindeys, or upon obstruction to the flow of urine through the ureters. Possibly on account of his obscure and confused notions on causation, the author sees fit to omit mention of the prophylactic value of the hot-water bath, as used in Vienna, and of the induction of premature labor. Possibly, also, for the same reason, Parvin advocates bleeding, an ancient measure, based on a false pathology, and long since condemned alike by clinical experience and responsible opinion."

I opine that that writer has rarely stood at the bedside of a puerperal patient in private practice in the rural districts, else he would not so confidently insist upon "the hot-bath as used in Vienna." For there he would not, in ninety-nine cases in a hundred, command a hot bath until his eclamptic patient had given up the ghost. "If puerperal convulsions are in a large majority of cases the expression of uræmia conditioned upon functional or organic disease of the kidneys upon the obstruction of the flow of urine through the ureters," why is it that, in the nine cases ot puerperal eclampsia which I have encountered, not a single patient has manifested, after the convulsions have ceased, any more renal abnormities, either organic or functional, than I have observed in normal labors?

If bleeding is an "ancient measure based upon a false pathology, and long since condemned alike by clinical experience and responsible opinion," why is it that timely bleeding has thus far been vindicated by my own happy results from the judicious practice of it? When, where and by whom, has more success been achieved in the treatment and prophylaxis of puerperal eclampsia, than by those who have always denied and still deny that uræmia, an occasional abnormal product of a diseased condition, is the prime and only cause of that fearful complication of the puerperal state?

So intensely interesting has been the treatment of puerperal eclampsia, that for two score years, it has been my habit to ask my numerous medical friends about their experience in the treatment of it, and I have yet to learn of any fatal case in which timely and free blood-letting was resorted to. Yet I have often heard of deaths consequent upon it, when the practitioner has failed to resort to timely venesection.

Some who condemn in toto the use of the lancet, never owned one nor ever performed the operation of phlebotomy. Of those adhering to the pathological theory that uræmia is the only prime or predisposing cause of puerperal eclampsia, who has suggested any means to quickly deplete the circulation of the claimed poisonous quantity of urea, that has ever wrought such fortunate results, as prompt and early venesection? Is it not time that obstetricians, upon whom rest the responsibility of preserving the lives of travailing mothers, enter a protest against the condemnation of venesection as a prophylactic precaution, in cases of threatened puerperal eclampsia, and as the foundation of the rational treatment of this terrible complication of the puerperal state, when it exists, since that condemnation is based upon irrational views of the pathology of the disease, flippantly iterated and reiterated by writers and lecturers, whose ratiocination pronounces a consequence of a disease a cause? In other words, they would have the practitioner "to put the cart before the horse," and thus face his burthen of responsibility in the lying-in chamber amid the wailings of the motherless, and the mourning of widowers, and others in whom the sufferings of parturient women excite the holiest sympathies, all in vain, because forsooth he has lent obedience to so called authority, in violation of his own common sense convictions?

So thoroughly am I convinced of the importance of blood-letting as the precedent means of successfully combating puerperal convulsions, that I regard the omission of it as not only irrational but also highly culpable. It is this conviction, arrived at by careful study and by successful experience, that has stimulated the writing of this paper in the interest of humanity in general, and the most interesting of all patients,—those who appeal to medical science and skill to conduct them safely through the dangers and travails of child-birth—those who, if science and skill be not at fault, ought always to survive the performance of the physiological function of reproduction.

If, Mr. Editor, some of the language used in this paper may seem harsh, and discourteous to some of our confreres, who are often spoken of as high authorities in medical matters, I have no apology to make for using it. In view of the humanity involved and the lives at risk, all of us should derive courage from our conscientious intelligent convictions and boldly assert them. It is high time, that the fashionable erroneous views of the etiology and pathology of puerperal couvulsions should be combated and exposed, and the malpractice based upon it condemned. *Non solitus jurare in verba magistri*, I have spoken in this paper according to the faith within me—a faith founded upon observation, experience and much thought,—and alone in behalf of much suffering women.

